# An Individual Cognitive Stimulation Therapy App for People With Dementia and Their Carers: Protocol for a Feasibility Randomized Controlled Trial

**DOI:** 10.2196/24628

**Published:** 2021-04-08

**Authors:** Harleen Kaur Rai, Justine Schneider, Martin Orrell

**Affiliations:** 1 Division of Psychiatry and Applied Psychology Institute of Mental Health University of Nottingham Nottingham United Kingdom; 2 School of Sociology and Social Policy Law and Social Sciences Building University of Nottingham Nottingham United Kingdom

**Keywords:** dementia, cognitive stimulation therapy, touchscreen technology, feasibility trial, quality of life, mHealth, apps

## Abstract

**Background:**

There is a need for more resources to support the cognition and quality of life of people with dementia. The individual cognitive stimulation therapy (iCST) app aims to provide cognitive stimulation and social interaction to people with dementia and carers through interactive touchscreen technology. The iCST app has been developed according to the principles of CST and iCST, which have previously shown to improve the cognition and quality of life of people with dementia and benefit the relationship between the person with dementia and his/her carer. The iCST app has also shown to improve the quality of the carer’s life.

**Objective:**

The aim of this study is to evaluate the usability of the iCST app intervention and the feasibility of conducting a full-scale randomized controlled trial (RCT) to assess the clinical effectiveness of the iCST app intervention compared to that of treatment-as-usual for people with mild-to-moderate dementia.

**Methods:**

We aim to recruit 60 people with mild-to-moderate dementia and their informal carers as dyads in a multi-center feasibility RCT with a treatment-as-usual control group. Both parties must be able to provide informed consent and participate in the intervention. Dyads will complete a baseline assessment that will include cognition and quality of life measures and they will subsequently be randomized (1:1) to the iCST app intervention in addition to usual care or to usual care only. All participants will be followed up at 5 weeks and at 11 weeks after the baseline assessments. A range of feasibility outcomes will be assessed, including recruitment and retention rates, intervention fidelity and usability, and acceptability of the outcome measures. A sample of the experimental group will be invited to a semistructured posttrial interview to further examine the experience of using the iCST app.

**Results:**

This study received funding in May 2015 and obtained ethical approval in March 2018. Data collection began in November 2018 and was completed in March 2020 with a total of 61 dyads recruited. Data analyses are in progress and the final results are expected to be available in the spring of 2021.

**Conclusions:**

This study will investigate whether it is feasible to conduct a full-scale RCT to evaluate the clinical effectiveness of the iCST app in comparison to that of usual care alone. In addition, this study will examine the usability of the iCST app. The data will provide information on potential modifications to be made to the intervention, study design, and study process.

**Trial Registration:**

ClinicalTrials.gov NCT03282877; https://clinicaltrials.gov/ct2/show/NCT03282877

**International Registered Report Identifier (IRRID):**

DERR1-10.2196/24628

## Introduction

### Background

Dementia poses a significant challenge to individuals in staying mentally stimulated and engaged. This is further exacerbated, given the lack of resources to support the cognition and quality of life (QoL) for people with dementia. Cognitive stimulation therapy (CST) is a nonpharmacological group treatment, which is strongly recommended by the National Institute for Health and Care Excellence. A previous randomized controlled trial (RCT) showed that CST can benefit the cognition and QoL of people with dementia [1]. The individual CST (iCST) intervention is delivered by a carer at home and has shown improvements in the relationship quality between the person with dementia and his/her carer and in the QoL of the carers [2].

Technology can improve accessibility to interventions by offering interventions on devices such as desktop computers and touchscreen tablets. For instance, in light of the COVID-19 pandemic wherein CST groups for people with dementia were unavailable, Cheung and Peri [3] found that it was feasible to offer virtual CST groups by using Zoom, a video conferencing software. This platform may support people with dementia to stay mentally stimulated and engaged in the safety of their homes. Touchscreen interventions, in particular, can have an array of benefits, as touchscreen platforms can be intuitive and therefore, have a high level of acceptance among people with dementia [4]. A systematic review has shown that touchscreen interventions with sound designs and tailored content can improve the well-being of people with dementia [5]. Moreover, these interventions can have a positive impact on the well-being of carers by decreasing their burden and improving the quality of the relationship with the person they are caring for by spending more time together. An example is the Computer Interactive Reminiscence and Conversation Aid (CIRCA) program, which offers computerized cognitive stimulation by prompting reminiscence among people with dementia supported by a wide range of multimedia stimuli on a touchscreen computer system. When used in a group setting, it can lead to improvements in both the cognition and QoL of people with dementia [6]. Researchers also found that CIRCA could positively benefit the relationship quality of the person with dementia and his/her carer when using the program together [7]. However, despite these encouraging findings, the field of computerized cognitive stimulation is underdeveloped [8], and high-quality studies involving carer-delivered computerized cognitive stimulation programs, in particular, remain scarce. More research in this field is warranted as the existing evidence seems to be promising; there are minimal risks in engaging with computerized cognitive stimulation and such interventions could potentially be cost-effective. Therefore, researchers have developed a novel, touchscreen iCST app, which aims to offer cognitive stimulation to people with dementia and can be used at home with carers. Rather than using a touchscreen computer system like CIRCA, the iCST app has been developed as a native app; thus, it can be downloaded from the App Store and Google Play stores on touchscreen tablets, which further improves the accessibility of the intervention. Like CIRCA, the iCST app aims to stimulate conversation between the person with dementia and his/her carer by using a range of topics, which can include reminiscence. However, the iCST app also offers other types of activities supported by multimedia stimuli in order to target specific cognitive functions such as memory and language. It is hoped that the iCST app may produce combined benefits of engaging in CST, iCST, and touchscreen technology. The app’s development followed the Medical Research Council Framework for developing complex interventions and the Centre for eHealth Research roadmap [9,10]. This included extensive reviews of CST and iCST materials and end-user involvement through informal consultations, focus groups, individual interviews, and usability questionnaires [11]. The next stage of development encompasses this feasibility RCT. Findings from this study will inform whether a large-scale RCT evaluating the clinical effectiveness of the iCST app is indicated by investigating relevant study parameters related to the study design and process.

### Study Aim

The aim of this study is to evaluate the usability of the iCST app intervention and the feasibility of conducting a full-scale RCT to assess the clinical effectiveness of the iCST app intervention compared to that of treatment-as-usual (TAU) for people with mild-to-moderate dementia.

## Methods

### Study Design

**Figure 1:**
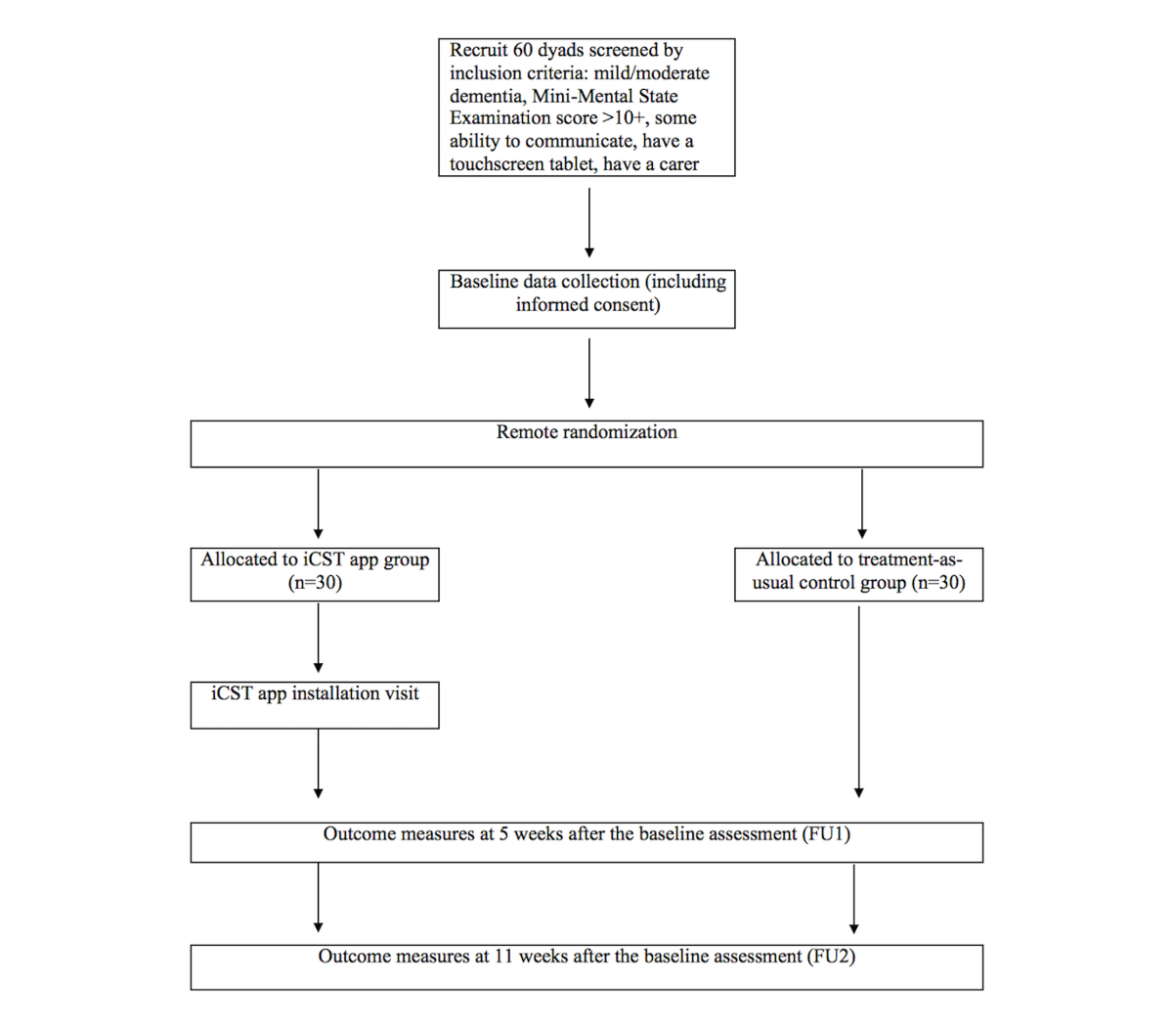
Flow diagram for the feasibility randomized controlled trial of the individual cognitive stimulation therapy app. FU1: first follow-up; FU2: second follow-up; iCST: individual cognitive stimulation therapy.

The 26-item CONSORT checklist of information to include when reporting feasibility trials will be used for this study to ensure that all the necessary and relevant information is reported [12]. This study was registered with ClinicalTrials.gov on July 19, 2017 (registration number: NCT03282877). This study is a multi-center, feasibility RCT with an allocation ratio of 1:1. People with dementia and carers will be recruited as dyads and will be randomized to either the experimental (completing 30-minute iCST app sessions twice or thrice per week) or the TAU control group for 11 weeks. Dyads will complete the baseline assessment prior to randomization and thereafter, the first follow-up will be completed at 5 weeks after the baseline assessments and the second follow-up at 11 weeks after the baseline assessments (Figure 1). A sample of the experimental group will be invited for semistructured posttrial interviews to gain insights into the acceptability of the iCST app, including the experience of using the app and any facilitators and barriers for implementation in daily life.

### Participants

Recruitment started on November 1, 2018 and is conducted in 5 secondary care settings in England: Derbyshire Healthcare National Health Service (NHS) Foundation Trust, Leicestershire Partnership NHS Trust, Lincolnshire Partnership NHS Foundation Trust, Northamptonshire Healthcare NHS Foundation Trust, and Nottinghamshire Healthcare NHS Foundation Trust. In addition, participants will be identified through a variety of settings, including general practitioner practices, community mental health teams, memory clinics, care homes, memory cafes, support groups, and voluntary sector organizations such as the Alzheimer’s Society. Remote recruitment will include registration on the website Join Dementia Research, publicizing the study using social media platforms such as Twitter, and the distribution of information leaflets and posters to organizations and professionals involved in the identification of possible participants.

### Eligibility Criteria

The sample will include people with mild-to-moderate dementia and their informal carers (relatives or friends). The inclusion and exclusion criteria were adapted from previous CST and iCST research studies [1,2] and are shown in Textbox 1.

**Inclusion and exclusion criteria for people with dementia and for their carers in this study.**

**Inclusion criteria**

For people with dementia

- Meet Diagnostic and Statistical Manual of Mental Disorders (DSM-IV) criteria for dementia [13].
- Score 10 or above on the Mini-Mental State Examination [14] or score of 16 or above on the Montreal Cognitive Assessment [15], where available.
- Some ability to communicate and understand (eg, ability to give informed consent).
- Ability to speak and understand English.
- See/hear well enough to participate.
- No major physical illness or disability affecting their participation.
- Age range: 50 years–no maximum age limit.
- Availability of a touchscreen tablet for the person with dementia and for the carer.
- Availability of a carer (or relative/friend) to participate in the activities.

For carers

- Minimum age: 21 years.
- Ability to speak and understand English.
- See/hear well enough to participate.
- No major physical illness or disability affecting their participation.

**Exclusion criterion for people with dementia and for carers**

Concurrent participation in any other interventional study.

### Sample Size

A formal sample size calculation is not appropriate for a feasibility trial. A previous audit of trials registered in the Clinical Research Network database in the United Kingdom found that most feasibility and pilot trials had a median of 30 or 36 participants per arm and the researchers recommend an upper limit of 60 participants for a feasibility trial [16]. Therefore, a target of 60 dyads is set for this study, leading to 30 dyads per treatment arm.

### Procedure

#### Screening for Eligibility

Anticipating logistical support from the National Institute for Health Research Clinical Research Network East Midlands, we expect that staff members at each research site will check the eligibility of referrals received from clinicians and staff at the recruitment sources. Participants fulfilling the inclusion criteria will be sent a participant information sheet containing full details about the study. If the dyad is interested in participating, they will be recruited into the trial and a date for the baseline assessment and consenting will be set by the Clinical Research Network staff member.

#### Randomization

Randomization will take place after consent and the baseline assessments by using a web-based central randomization service called Sealed Envelope [17]. Block randomization will be employed with block sizes of 4 to 6 (randomly varied and generated by Sealed Envelope), which is a useful method for small sample sizes to allocate an equal number of participants to each treatment arm [18]. The researcher at the local site will perform each randomization using the participant identification code of the person with dementia only. The allocation to the experimental or TAU control group will automatically apply to the carer as well. Dyads will be informed of their allocation outcome over the telephone and, if necessary, a visit will be arranged for dyads in the experimental group to install the iCST app.

#### Blinding

This trial will include both blinded and unblinded researchers at each local site. It is not possible to blind the participants to their treatment arm as the iCST app is a nonpharmacological intervention. However, each study site will include at least one researcher kept in ignorance of study allocations. The baseline assessment can be performed by either researcher. However, both follow-ups will be completed only by the researcher who is unaware of the randomization outcome for each dyad. If disclosure does occur, this will be recorded by the visiting researcher along with details on how it occurred. The unblinded researcher will perform the randomization, communicate the outcome with the participants, and for the experimental group, install the iCST app, provide weekly telephone support calls, and complete the usability and acceptability questionnaire at the end of the study. Furthermore, the unblinded researcher will not be informed about the results of the assessments.

### iCST App Intervention

Participants in the experimental group will use the iCST app (prototype v3.0) over 11 weeks after the baseline assessments. The content of the app was modified from the paper-based iCST manual, including the principles, themes, and activities [2], and was based on consultations and qualitative research with people with dementia, carers, and the software development company [11]. The iCST app is a one-to-one, carer-led, home-based program of structured cognitive stimulation for people with dementia but delivered on a touchscreen tablet. It includes 21 activities consisting of both game-like interactive features such as audio-visual stimuli and discussion questions (Table 1). These activities offer mental stimulation not only through the content on the app but also through conversation.

**Table 1:** List of the individual cognitive stimulation therapy app activities (prototype v3.0).

Type of activity	Activity name
Games only	Being Creative Spaceman Trivia Quiz Word Search Sudoku Being Active Brainstorm
Discussion questions only	Past Events Useful Tips My Life Arts Old Wives’ Tales Toys Are Us
Games and discussion questions	Sounds Odd one Out The Price is Right Globe Trotter Food In Pairs Sayings ISpy

**Figure 2:**
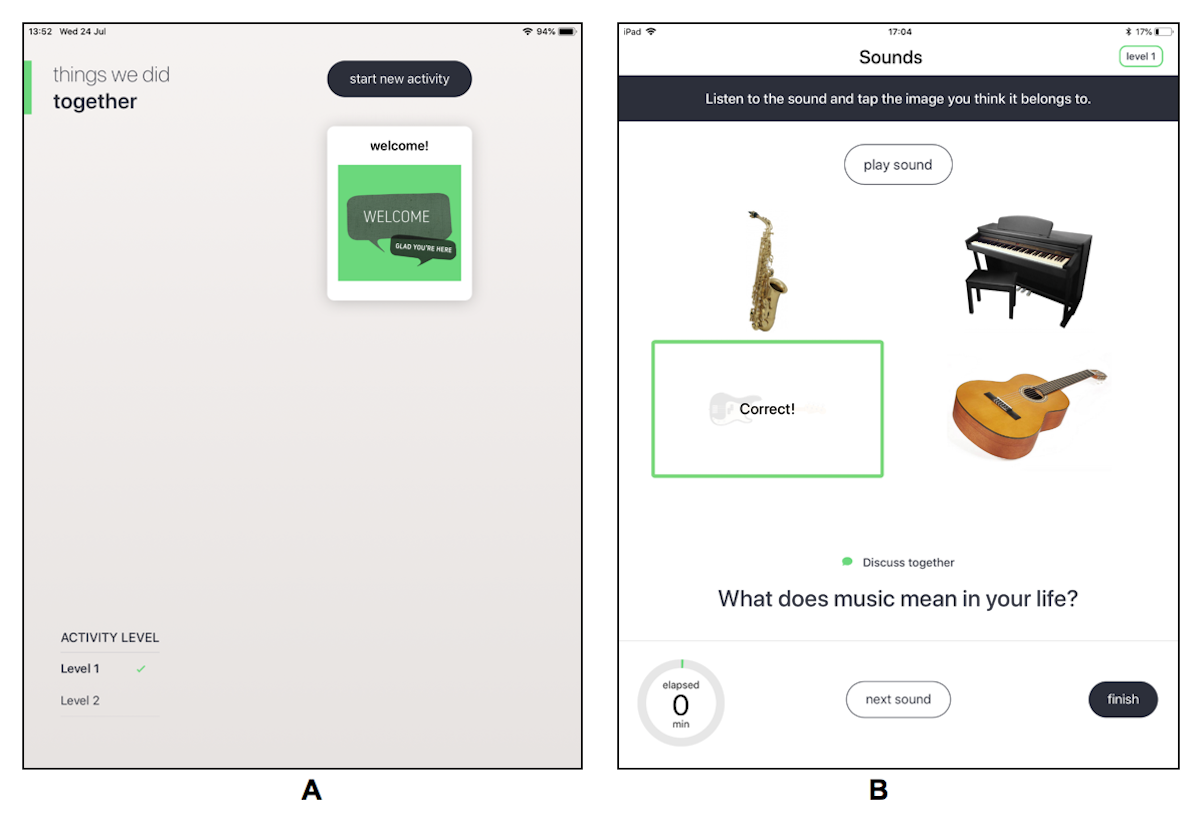
Screenshots of the individual cognitive stimulation therapy app. A. home screen and B. sounds activity.

In addition, this app includes several other features such as a short introduction section explaining the background and key tips for using the app, a home screen that features completed activities, and a choice of 2 levels. Level 2 contains either more challenging content or different questions from Level 1, and it is up to the participants to determine which level they feel more comfortable with for each activity. Figure 2 contains screenshots of the iCST app. Considering previous CST and iCST research and findings from the development work, it is recommended that participants use the app for 2 or 3 times a week for 30 minutes [2]. Participants are free to spend more time on the app if they wish, and this will be recorded during the weekly telephone calls.

### Training and Adherence

In order to ensure treatment integrity for all participants, individual study sites will receive a demonstration of the iCST app along with training in its installation and use prior to the start of recruitment. Dyads in the experimental group will receive an in-home visit from the unblinded researcher who will install the app and explain how it works using a short supplementary document containing instructions with screenshots of the app. Furthermore, all dyads will receive weekly telephone support calls from the same unblinded researcher in order to monitor adherence and to track the overall progress and any challenges or technical difficulties with using the iCST app. Phone calls will be completed with the carer and all questions will be included on a standardized telephone sheet. The questions relate to general experience, number of sessions completed in a week (on average), amount of time spent per session (on average), enjoyment, and any likes/dislikes. Any reason for not being able to use the iCST app over the week will also be recorded on the telephone sheet.

### TAU Group

The control group will consist of the TAU group and will not receive any additional interventions. TAU groups are typically used to compare experimental interventions to care, which participants already receive in practice [19]. Therefore, the TAU control group will enable us to compare the effects of the iCST app with the natural progression of people with dementia under conditions of usual care. The treatments and services that are already available to people with dementia and their carers randomized to the TAU control group may differ between and within recruitment sites, for instance, regarding acetylcholinesterase inhibitors or being involved in some form of cognitive stimulation already. However, it is unlikely that people have access to computerized versions of iCST since these do not exist, to the best of our knowledge. The visiting researcher will record any current participation with CST groups and use of acetylcholinesterase inhibitors at the baseline assessment.

### Outcomes

#### Feasibility Outcomes

In order to determine the feasibility of conducting a full-scale RCT with the iCST app in the future, this trial will investigate key feasibility aspects, including the rates of recruitment, screening, randomization, and retention by using enrolment logs [9]. Acceptability of the outcome measures will be evaluated by assessing the completion rates, and the acceptability and fidelity of the iCST app will be evaluated through weekly telephone support calls, analytics, and a usability and acceptability questionnaire. It is expected that >75% of the participants in the experimental group will need to complete the recommended minimum of 2 activities on average every week for the iCST app to be considered feasible. This benchmark has been adopted based on work in some previous feasibility trials, including psychological treatments where benchmarks for successful adherence ranged between 75% and 80% [20,21].

#### Clinical Outcomes

Previous CST and iCST research and the Interdem consensus statement on outcome measures for dementia informed the outcome measure selection for this study [22]. Key outcome measures of interest for people with dementia (Textbox 2) are cognition and QoL, as previous CST research has shown improvements in these domains [1]. For carers, the key outcome measure (Textbox 3) is QoL as previous iCST research has shown improvement in the QoL of carers [2]. This study additionally includes technology-related scales to assess the usability and acceptability of the iCST app and computer use self-efficacy (Textbox 4). All assessments will take place in the homes of the participants. Wherever possible, 2 researchers will visit the participants in order to interview the person with dementia and his/her carer separately. It will be possible to conduct the assessments over 2 days in case of fatigue or other practical issues such as lack of time. As in the previous iCST trial, the first follow-up at 5 weeks will be included to safeguard data against loss-to-follow-up [2]. The second follow-up will take place at 11 weeks after the baseline as this should be the point that participants in the experimental group will have completed each activity on the iCST app.

**Outcome measures for people with dementia.**

**Measures**

- Cognition measured using the Alzheimer’s Disease Assessment Scale-Cognitive Subscale [23].
- Quality of life measured using the Quality of Life-Alzheimer’s Disease (QoL-AD) questionnaire [24]. Carers will complete the family version of the QoL-AD.
- Health-related quality of life measured using the EuroQoL 5-dimension questionnaire [25].
- Relationship quality measured using the Quality of the Carer-Patient Relationship questionnaire [26].
- Symptoms of depression measured using the Cornell Scale for Depression in Dementia [27]. Carers will complete this questionnaire as an informant.
- Behavioral disturbances measured using the Neuropsychiatric Inventory and will be rated by the carer only [28].
- Functional abilities measured using the Bristol Activities of Daily Living Scale [29] and rated by the carer only.

**Outcome measures for carers.**

**Measures**

- Health-related quality of life measured using the EuroQoL 5-dimension questionnaire [25].
- Anxiety and depression measured using the Hospital Anxiety and Depression Scale [30].
- Relationship quality measured using the Quality of the Carer-Patient Relationship questionnaire [26].

**Technology scales for people with dementia and carers.**

**Scales**

- Self-efficacy beliefs in computer/tablet use measured at baseline only using the Computer User Self-Efficacy scale [31].
- Usability and acceptability of the individual cognitive stimulation therapy app measured at the second follow-up and in the experimental group only by using the Questionnaire of Usability and Acceptability [32].

### Posttrial Interviews

A small proportion of dyads in the experimental group will be invited to participate in joint semistructured interviews. The purpose of the interviews is to gain additional information on the layout and content of the iCST app, the overall experience of using it as a dyad, and any practicalities surrounding its use in daily life. The interview serves as a complementary data collection method to the weekly telephone support calls and the usability questionnaire, as a semistructured interview generates more in-depth data that otherwise cannot be accessed through quantitative methods only [33]. A discussion guide will be developed, including the key areas mentioned before, to help guide the interview. Each dyad in the experimental group will be invited to participate in the interview upon completion of the study. If they are interested, further details for the person with dementia and carer will be sent. If a dyad agrees to participate, a date for the interview will be set. All interviews will take place in the home of the participants. Written informed consent will be obtained from both participants by the unblinded researcher. The data will be audio-recorded, transcribed, and subsequently stored on a password-protected computer at the University of Nottingham.

### End of Study

The second follow-up constitutes the end of the study for participants. At this final visit by the blinded researcher, all participants will be given a £10 (US $13.01) App Store or Google Play store voucher in order for them to download the iCST app once it has been released on the app stores. This will be accompanied by an instructional document on how to redeem the voucher and a newsletter containing information on what will happen next, such as making improvements to the app and analyzing and disseminating the results.

### Ethical Considerations

Ethical approval has been obtained from the Yorkshire and the Humber-Bradford Leeds Research Ethics Committee and NHS Health Research Authority in March 2018 (reference number 17/YH/0405).

### Consent

People with mild-to-moderate dementia will be recruited in the study and are expected to be able to give informed consent for participation, provided that appropriate care is taken in explaining the research and sufficient time is allowed for them to reach a decision. Written informed consent will be taken at baseline from both the person with dementia and the carer. Since the intervention requires joint participation, it is likely that both participants will consult each other in making their decision. Therefore, it is possible that any individual participant’s decision to either participate or not participate with the research may be influenced by the other participant. However, it is important that individual participants are not forced to make a decision against their will and the researcher will spend as much time as necessary in speaking to the participants individually about the research. It will be made clear to both people with dementia and carers that no disadvantage will accrue in terms of the current care they receive or any future research opportunities if they choose not to participate or withdraw from the study. The consent form will be signed and dated by the participant and the researcher before they enter the study. One copy will be given to the participant and one will be retained at the local study site.

### Adverse Events

Previous work with CST, maintenance CST, and iCST has not documented any harmful side effects nor any serious adverse events from participating in the intervention activities [1,2,34]. Given that the iCST app is based on the principles of CST and follows a comparable structure to iCST, it is expected that this study will not lead to harmful side effects for either the person with dementia or the carer. Researchers will be made aware of any adverse events during follow-up assessments or the weekly telephone support calls. The trial manager and chief investigator of the study will be informed in case of any adverse events, and they will assess the severity of the adverse event. Serious adverse events include death, illness related to a previous health condition, or hospitalization.

### Data Security and Entry

Each study site will create their own password-protected spreadsheet containing participant identifiable information and allocation outcome for the dyad. This spreadsheet can only be accessed through a secure NHS Trust computer. After collection of the data from each site, the data will be stored in a secure cabinet at the University of Nottingham. Identifiable information, including the consent forms, will be kept in a separate locked cabinet. After reviewing the data and checking the scoring, it will be entered manually into SPSS version 25 (IBM Corp) for Windows, which will be used for all the analyses.

### Statistical Analyses

Key feasibility outcomes will be reported through frequencies and will include the number of participants screened, recruited, randomized, and retained through the duration of the trial. Adherence to the intervention will be assessed by calculating the average number of iCST app activities completed by the dyad logged in the weekly telephone calls. The usability and acceptability of the iCST app will be further investigated by examining data from the weekly telephone calls, posttrial interviews, and by calculating scores on the Questionnaire of Usability and Acceptability, with higher scores indicating higher levels of usability and acceptability. Data from the posttrial interviews will be coded and summarized and may be analyzed thematically with specialized software if sufficient data have been obtained to reach data saturation [35]. Lastly, outcome measures will be assessed for appropriateness by calculating missing data rates within the measures and across the follow-ups. As this is a feasibility trial and null hypothesis significance testing is inappropriate due to a likely lack of power to detect significant effects of the intervention [36-38], analyses will mainly include descriptive statistics computed for each group and outcome measures, including means, standard deviations, 95% confidence intervals, and effect sizes [12,39]. However, in order to compare the outcomes on each of the questionnaires between the 2 groups, an analysis of covariance will be undertaken, which will help to better understand any trends in the data. All analyses will be based on the intention-to-treat principle in that all available data will be included in the analyses. Rules for missing data will be adapted from the main iCST trial [2]. Data will not be imputed if outcome measures or assessments are missing in full, and imputation (using prorating) will only be used when fewer than 20% of cases are missing on any given measure.

## Results

Funding for this study was obtained in 2015. Development work for the iCST app lasted 1 year and took place between October 2017 and October 2018 [11]. Data collection for the feasibility RCT started in November 2018; however, due to the COVID-19 pandemic, recruitment had to be terminated prematurely. Data collection was completed in March 2020. Data cleaning, entry, and analyses are underway. The full results of this study are expected in the spring of 2021.

## Discussion

### Overview of This Study

The iCST app is the first computerized version of iCST, which can be used on touchscreen tablets by people with dementia and their carers. It aims to provide mental stimulation and to stimulate conversation between dyads through the use of interactive touchscreen technology. Based on previous research, it is expected that regular use of the iCST app could potentially lead to improved cognition and QoL for the person with dementia and his/her carer. This is an innovative feasibility RCT that sets out to evaluate the feasibility of conducting a full-scale RCT with the iCST app compared to a TAU control group and to assist the development of a protocol for a full-scale trial. A range of data will be collected on relevant study-related aspects for a potential full-scale RCT, including the study design and process and the feasibility and usability of the iCST app. Data collection is supported by a mixed-methods approach where quantitative data from questionnaires and analytics will be complemented by qualitative data from telephone calls and interviews with people with dementia and carers. A few challenges in the recruitment process are anticipated, given that the iCST app is only compatible with certain touchscreen tablets and software versions and requires internet access at home, which may lead to the exclusion of dyads not meeting these criteria. These can be overcome by ensuring an appropriate timeframe for recruitment supported by adequate resources in terms of capacity at study sites. The findings from this feasibility RCT will be used to draw recommendations in terms of conducting a full-scale trial and determine which modifications are necessary. This will be done using the Acceptance Checklist for Clinical Effectiveness Pilot Trials, which consist of several trial components ranging from trial design and interventions to randomization and data procedures [40]. It will be used to determine which components of the trial will need amendments and how this can be achieved. In addition to a large-scale RCT with the iCST app, other research activities could consist of an implementation study, which would investigate the cost-effectiveness and the accessibility of the iCST app among people with dementia from varying backgrounds. This could include consultation work with people with dementia and carers in order to explore facilitators and barriers toward accessing technology, the iCST app, and its use.

### Conclusions

This study will give insights into the feasibility of conducting a full-scale RCT with the iCST app compared to a TAU control group. The full results of this feasibility RCT, including data on the intervention in terms of usability and adherence and outcome data are expected in early 2021. These results will inform whether a full-scale RCT is feasible and which modifications to the study design and process and intervention are needed.

## Abbreviations

CIRCA: Computer Interactive Reminiscence and Conversation Aid
CST: cognitive stimulation therapy
iCST: individual cognitive stimulation therapy
NHS: National Health Service
QoL: quality of life
RCT: randomized controlled trial
TAU: treatment-as-usual
